# Can UK NHS research ethics committees effectively monitor publication and outcome reporting bias?

**DOI:** 10.1186/s12910-015-0042-8

**Published:** 2015-07-25

**Authors:** Rasheda Begum, Simon Kolstoe

**Affiliations:** Institute of Biomedical and Biomolecular Science, University of Portsmouth, King Henry Building, Portsmouth, PO1 2DY UK

**Keywords:** Publication ethics, Reporting bias, Publication bias, Research ethics, Ethics committees

## Abstract

**Background:**

Publication and outcome reporting bias is often caused by researchers selectively choosing which scientific results and outcomes to publish. This behaviour is ethically significant as it distorts the literature used for future scientific or clinical decision-making. This study investigates the practicalities of using ethics applications submitted to a UK National Health Service (NHS) research ethics committee to monitor both types of reporting bias.

**Methods:**

As part of an internal audit we accessed research ethics database records for studies submitting an end of study declaration to the Hampshire A research ethics committee (formerly Southampton A) between 1st January 2010 and 31st December 2011. A literature search was used to establish the publication status of studies. Primary and secondary outcomes stated in application forms were compared with outcomes reported in publications.

**Results:**

Out of 116 studies the literature search identified 57 publications for 37 studies giving a publication rate of 32 %. Original Research Ethics Committee (REC) applications could be obtained for 28 of the published studies. Outcome inconsistencies were found in 16 (57 %) of the published studies.

**Conclusions:**

This study showed that the problem of publication and outcome reporting bias is still significant in the UK. The method described here demonstrates that UK NHS research ethics committees are in a good position to detect such bias due to their unique access to original research protocols. Data gathered in this way could be used by the Health Research Authority to encourage higher levels of transparency in UK research.

## Background

Reporting bias occurs when the decision of how to publish a study is influenced by the direction of its results [1]. It is a well-recognized issue [2] that has recently come to the forefront of the public agenda in the UK due to the activities of the international “AllTrials” petition [3], and the writings of populist science authors such as Ben Goldacre [4]. Authoritative academic analyses have been conducted through the long-running work of organizations such as the Cochrane Collaboration and British Medical Journal (among others) who continue to generate significant public and media pressure [5].

In December 2011 the Health Research Authority (HRA) was established in the UK to protect and promote *“the interests of patients and the public in health research”* [6]. As part of this remit the HRA was challenged to formulate proposals to improve transparency in health research [7]. Since the HRA incorporates the National Research Ethics Service (NRES), whose research ethics committees (RECs) review and provide ethical opinions on all research using NHS patients, it seemed logical that the HRA plays a greater role in monitoring publication outcomes. In recognition of this the HRA business plan for 2013–2014 contains the stated aim of *“working with all the relevant partners to help create an environment where clinical trials are registered and research results get published”* [8].

To investigate the logistics and effectiveness of NHS research ethics committees monitoring reporting bias we have determined the publication status of all projects submitting an end of study declaration to a single research ethics committee over a defined timeframe. We have also looked for discrepancies between outcomes stated in the original ethics application and those reported in the final academic papers.

## Methods

### Cohort of studies

As part of an internal audit we accessed Integrated Research Application System (IRAS) records of research projects that submitted an end of study notification to the South Central, Hampshire A research ethics committee (formally Southampton A) between 1st January 2010 and 31st December 2011. Studies were stratified from definitions in the REC forms (see Table 1).

**Table 1:** Characteristics of studies and publication status

	Publication status of all studies (n = 116)			Reporting outcomes of published studies (n = 28)		
	Published 37 (32 %)	Unpublished 79 (68 %)	Total 116	Studies with discrepancies 16 (57 %)	Studies consistent with IRAS form 12 (43 %)	Total 28
Study type						
Clinical trial	10 (27 %)	27 (73 %)	37 (32 %)	3 (75 %)	1 (25 %)	4 (14 %)
Clinical Investigation	2 (25 %)	6 (75 %)	8 (7 %)	1 (50 %)	1 (50 %)	2 (7 %)
Questionnaire/mixed methodology	13 (57 %)	10 (43 %)	23 (20 %)	8 (62 %)	5 (38 %)	13 (46 %)
Qualitative study	3 (33 %)	6 (67 %)	9 (8 %)	2 (67 %)	1 (33 %)	3 (11 %)
Tissue or data	4 (27 %)	11 (73 %)	15 (13 %)	1 (25 %)	3 (75 %)	4 (14 %)
Other	1 (13 %)	7 (87 %)	8 (7 %)	1 (100 %)	0 (0 %)	1 (4 %)
Unknown study type	4 (25 %)	12 (75 %)	16 (14 %)	0 (0 %)	1 (100 %)	1 (4 %)
Type of sponsor						
NHS or HPSS	16 (35 %)	30 (65 %)	46 (40 %)	7 (50 %)	7 (50 %)	14 (50 %)
Academic	13 (38 %)	21 (62 %)	34 (29 %)	7 (58 %)	5 (42 %)	12 (43 %)
Industry	3 (12 %)	21 (88 %)	24 (21 %)	2 (100 %)	0 (0 %)	2 (7 %)
Unknown sponsor	5 (42 %)	7 (58 %)	12 (10 %)	N/A	N/A	
Number of centres						
Single centre	11 (70 %)	26 (30 %)	37 (32 %)	7 (63 %)	4 (36 %)	11 (39 %)
Multi-centre	21 (33 %)	42 (67 %)	63 (54 %)	9 (53 %)	8 (47 %)	17 (61 %)
Unknown	5 (31 %)	11 (69 %)	16 (14 %)	N/A	N/A	
Sample size						
<100	14 (29 %)	34 (71 %)	48 (41 %)	7 (50 %)	7 (50 %)	14 (50 %)
≥100	14 (30 %)	33 (70 %)	47 (41 %)	9 (63 %)	5 (36 %)	14 (50 %)
Unknown sample size	9 (43 %)	12 (57 %)	21 (18 %)	N/A	N/A	
Number of papers						
1	N/A	N/A		11 (58 %)	8 (42 %)	19 (68 %)
>1	N/A	N/A		5 (56 %)	4 (44 %)	9 (32 %)

Percentages may not add up to 100 due to rounding. Stratification taken from the REC application form with “unknown” referring to either a missing form or data missing from the application

### Literature search

Publications were located through a literature search in three bibliographic databases: Web Of Science, PubMed and Google Scholar. Search queries used the chief investigator’s last name and keywords from the study title. The literature search began in October 2013 and additional new publications monitored until August 2014. A publication was defined as a peer-reviewed paper published in an academic journal. Further analysis was carried out on clinical trials and Clinical Trial of an Investigational Medicinal Product (CTIMPs) to see if they had been registered (and had initial results published) on ClinicalTrials.gov, the European Clinical Trials Register, or isrctn.org. These searches used the sponsor’s protocol number, the European Clinical Trials Database (EudraCT) number, the International Standard Randomised Controlled Trial number (ISRCTN) and/or keywords from the study title. Researchers were not contacted directly so as to determine the level of information that could be gained from the publicly available databases alone.

### Outcome reporting bias

Outcome reporting was examined by comparing primary and secondary outcomes originally stated in the REC application with those reported in the final publications. Discrepancies were divided into three categories: 1) a missing outcome not reported in the final publication, 2) change of an outcome from primary to secondary (or vice versa), 3) addition of an outcome in the final publication.

### Data analysis

Data was analysed using descriptive statistics and odds ratios calculated using MedCalc 13 [9].

### Ethics and data-access

Ethical approval was not required from a statutory committee for this work. Raw data is not available due to the researchers unique access to the research ethics database as members of a REC.

## Results

### Included studies

A total of 116 studies were included in this study. As the inclusion criteria were based upon end of study notifications, original ethics applications were dated between 2003 and 2011. Characteristics of the included studies are shown in Table 1. The type of study design was unclear for 16 studies, the type of sponsor unknown for 12 studies, the number of centres unknown for 16 studies and the sample size unknown for 21 studies due to lack of information in the REC application. Thirty studies in the cohort contributed towards an educational degree.

### Publication outcomes

**Fig. 1:**
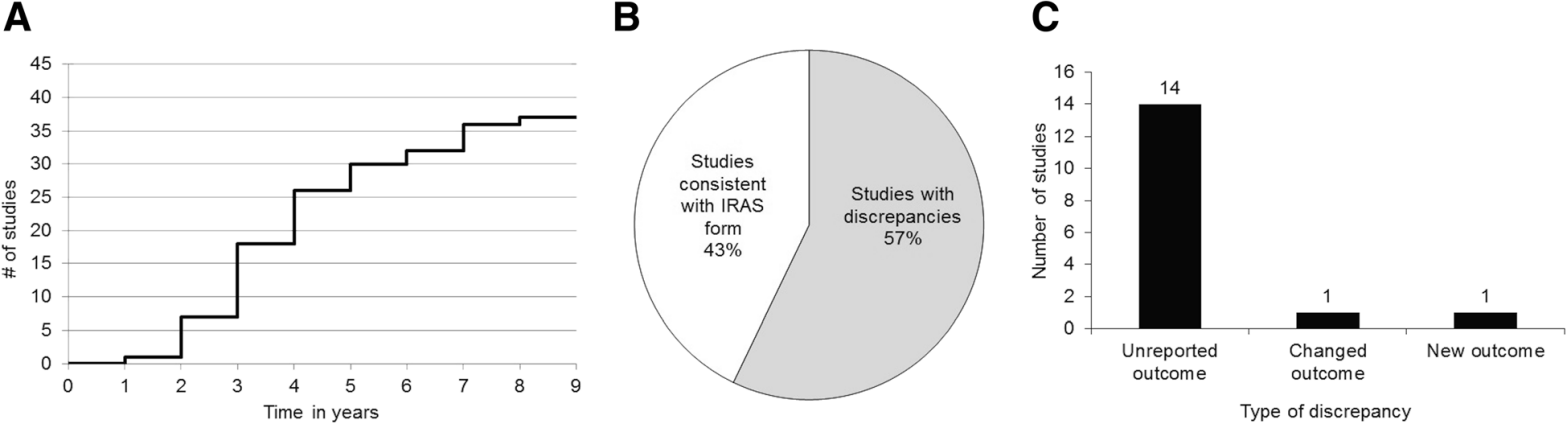
**a** Time taken between initial ethics submission and the first publication for the 37 studies that published. **b** Percentage of published studies that were consistent with IRAS form (n = 28). **c** Types of discrepancies identified between initial application and final publication

The literature search identified 57 publications for 37 studies giving an overall publication rate of 32 %. Twenty-one studies had been presented as a conference abstract, 11 of which also had a journal publication. A conference abstract alone was not considered a publication in line with previous studies due to the lack of information inherent in this type of report [10]. Twenty-five studies had more than one publication (range 1–6). Industry sponsored studies were significantly less likely to be published than studies sponsored by academic institutions, the NHS or Health and Personal Social Services (HPSS) sponsored studies (OR = 0⋅25, p = 0 · 04, CI 0⋅07-0⋅92). Attempts to stratify the data in other ways produced no significant results using simple odds-ratio calculations. Of the 37 studies that published at least one paper, the mean time for publication (from date of ethics submission) was 3⋅9 years with the highest number of studies (11) publishing between 3 and 4 years (Fig. 1a). Of 37 clinical trials in this cohort, 26 were registered on the ClinicalTrials.gov website with five (19 %) having posted summary results. Twenty-two out of 27 CTIMPs were registered through EudraCT with only one of the missing five on ClinicalTrials.gov. Only 5 of the 37 clinical trials were registered on the ISRCTN registry, four of which were on ISRCTN only and one on all three databases. This gave 7 out of 37 clinical trials (with 4 CTIMPs) not registered on any of the three main trial registration databases, although these trials had commenced prior to the requirement for registration.

### Consistency of outcomes

Full REC applications were available for twenty-eight studies for analysis of outcome-reporting bias (Table 1). A total of 141 outcomes were identified across the 28 studies from the original REC applications. These included 50 primary outcomes and 91 secondary outcomes. The median number of primary outcomes was 1 (range 1–6) and the median number of secondary outcomes was 3 (range 1–11). Four studies did not have any secondary outcomes stated whilst twelve studies had more than one primary outcome. Out of the 141 planned outcomes 32 (23 %) were missing in the final publications, including 6 unreported primary outcomes. Twelve studies (43 %) were entirely consistent with their original REC form whilst 16 (57 %) studies had discrepancies (Fig. 1b). Fourteen studies had missing outcomes including 3 clinical trials, 8 questionnaire/mixed methodology studies and 2 qualitative studies. One study categorised as “other” reported a statistically significant secondary outcome as a primary outcome (Fig. 1c). One tissue study had a new subgroup analysis in the publication that was not mentioned in the REC form.

Amongst the studies with unreported outcomes, six studies had at least one unreported primary outcome and thirteen studies had at least one unreported secondary outcome. The median proportion of unreported outcomes per study was 38 % (CI 25 %-75 %). There was a weak correlation between the number of papers a study produced and the percentage of reported outcomes (p = 0·94). There was no correlation between the number of outcomes and the percentage of reported outcomes (p = 0·62).

## Discussion

If the reporting of research is a fundamentally ethical issue [11–13] it seems well within the remit of research ethics committees to encourage researchers to commit to publishing results as a condition of ethical approval as well as monitoring subsequent publications [14]. Although it is appreciated that research can be reported in a number of different ways, this study chose to only look at the peer-reviewed scientific literature as this is the main source of information for the expert community who are best placed to utilise research outcomes. The publication rate of 32 % is consistent with previous studies conducted by research ethics committees that have found publication rates in the range of 20 %-76 % [15–18]. However, unlike many countries where such studies can only really provide snapshots from individual hospitals or regions, extending the method described here across the whole HRA ethics system could potentially capture national data and perhaps provide a continuous monitoring service for the UK.

The main weakness of this study was the short time available to researchers for data analysis and publication (a maximum of three years since end of study notification), especially as the literature seems to show that especially clinical drug trials publish on average between 4 and 8 years after a study [14]. However, this widely quoted timeframe is ambiguous because it is unclear whether it is measured from when the studies started or when they were completed. If the original date of submission to the Hampshire A research ethics committee is used, the studies analysed in this paper had between 2 and 10 years to publish at least some sort of peer reviewed result or commentary. It may be hoped that the percentage of projects publishing might improve as time goes on, but the data did show a mean time of 3⋅9 years between initial ethics application and publication for the studies that did publish (Fig. 1a), suggesting that the timeframe used here is not unreasonable. Other weaknesses included limiting this study to a single research ethics committee and not contacting researchers to determine reasons for non-publication. However, the purpose of this study was to determine a simple method that could reasonably be applied by individual RECs without extensive research funding.

Attempts to stratify the studies did not give significant p values for most study characteristics based upon a simple odds ratio calculation [19]. The only significant difference was with studies sponsored by industry that only showed a 12 % publication rate, compared to around 30 % for other types of sponsors. The reason for this cannot be determined here, but is consistent with the perception that industry suppresses results [20].

Unreported outcomes were the main reporting discrepancy identified between original REC applications and final publications. The figure of 57 % overall discrepancy between the originally planned outcomes and those reported in the final application is consistent with previous literature where discrepancies in reporting of primary outcomes were found in 62-66 % of clinical trials [21, 22]. Again conducting this study from within the HRA system proved particularly powerful as original REC application forms could be analysed. However, administrative changes between 2003 and the current time meant that electronic copies of REC forms were only available for a small number of studies, whilst some paper REC forms had already been discarded, so not all published studies could be included in this analysis. It was also not clear whether additional information from all study amendments had been located.

## Conclusion

The results described here demonstrate that NHS research ethics committees are able to effectively monitor publication and outcome reporting bias. This is significant because these committees hold complete records of all human research that is subject to certain legal regulation or conducted within the NHS. This places them in a stronger position than individual sponsors or research funders when it comes to auditing or monitoring reporting bias. However, such monitoring is not currently part of their remit. At the moment committees are composed of up to eighteen volunteer members and one REC manager, reviewing approximately 45 full applications per year. Although this project included just a subset of studies from one committee, it occupied a full-time masters student for most of a year. This would have significant resource implications on RECs and the HRA if comprehensive monitoring identical to this study was to be carried out for all studies submitted to all 68 committees currently overseen by the HRA. An alternative might be to conduct smaller studies such as described here on a regular basis, not for policing researchers, but rather to allow the monitoring of various ways to encourage researchers to publish both their projects as a whole and the outcomes they had originally committed to measure.
